# Association of tuberculous thyroiditis and papillary carcinoma of the thyroid: a rare coincidence

**DOI:** 10.11604/pamj.2014.19.118.4805

**Published:** 2014-10-01

**Authors:** Noureddine Errami, Amine Benjellounb, Bouchaib Hemmaouia, Karim Nadoura, Fouad Benariba

**Affiliations:** 1 ENT Unit, Mohammed V Military Hospital, Rabat, Morocco; 2 Pulmonology Unit, Avicenne Military Hospital, Marrakech, Morocco

**Keywords:** Thyroid, tuberculosis, papillary carcinoma

## Abstract

We report the case of a 25 year-old patient with no medical history, admitted to our unit for nodular goiter of the right lobe without clinical or laboratory signs of hyperthyroidism. We carried out a right lobo-isthmectomy revealing the association of tuberculosis and thyroid papillary carcinoma. A left lobectomy has, therefore, been performed in a second stage. The patient underwent a six-month antituberculosis treatment with a good clinical outcome. We discuss this rare association and its best diagnostic and therapeutic support, with a review of the literature.

## Introduction

Thyroid is a rare location of tuberculosis even in the endemic countries [1]. The frequency of this disease is about 0.1- 0.4% of all tuberculous locations [2]. The association of tuberculosis and papillary carcinoma in the same thyroid is exceptional [3]. We report a case of this rare coincidence and discuss both diagnosis and treatment.

## Patient and observation

**Figure 1:**
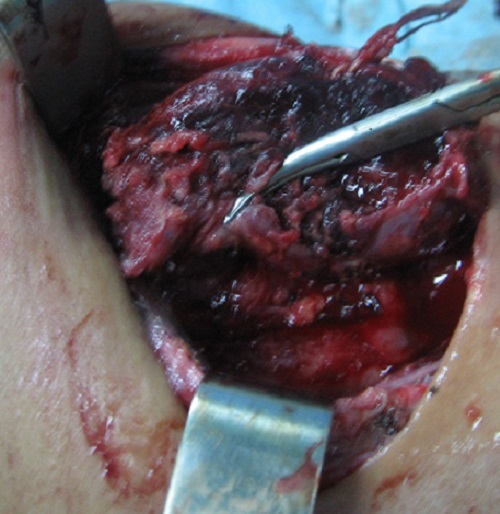
Necrosis of the right thyroid lobe

**Figure 2:**
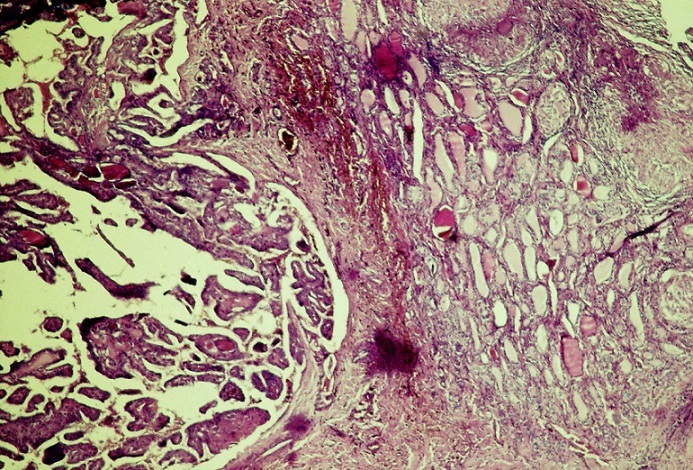
Microphotograph showing the association within the thyroid parenchyma (middle) of papillary carcinoma (left) and granuloma with epithelio-gigantocellular process and caseous necrosis (HEx10)

A 25 year old patient with no medical history showed a 5 cm basicervical anterior swelling, lateralized in the right, which appeared 4 months earlier. The swelling was associated to a mild dysphagia, without evident weight loss or infectious signs. Cervical ultrasound revealed a hypertrophy of the right thyroid lobe, with a 4 cm nodular cyst of the apex and a 1.5 nodule of the base. Biology was normal with no inflammatory signs. Thyroid function tests were normal. The patient was admitted in the ENT unit for right lobectomy. Scintigraphy and fine needle aspiration cytology (FNAC) were not performed before surgery. At the opening of the center line of the neck, we were surprised to find caseum coming from the thyroid with a necrotic destruction of the right lobe (Figure 1), frozen section was not at disposal. The right Loboisthmectomy and dissection of the recurrent nerve were very difficult. Histology showed an epithelioid and gigantocellular granulomatous process with caseous necrosis of the thyroid associated with a 1.5 cm papillary carcinoma (Figure 2).

HIV serology was negative and general check-up looking for other locations of tuberculosis revealed no abnormalities (Chest X ray, sputum analysis, abdominal ultrasound). A new surgery was performed afterward for left lobectomy. Histology revealed no tuberculous or neoplastic disease. The patient received a six-month antituberculosis regimen: 2RHZE/4RH (R:Rifampicin 10 mg/ kg/ j, H:Isoniazid 5 mg/ kg/ j, Z:Pyrazinamid 20-30 mg/ kg/j), with a satisfying uneventful evolution. No complementary anticancer treatment was necessary.

## Discussion

Thyroid tuberculosis was first described by Lebert in 1862. It is very rare and found in 0.1 to 1% of reported cases of tuberculosis [1]. It represents 0.6 to 1.15% of FNAC performed for solitary thyroid nodule in some endemic areas (India) [4]. All age groups can be affected, with an average age between 30 and 46 years [4] and a female predominance. The authors emphasize that the relative strength of the thyroid to the BK, due to its good oxygenation (rich vascularization) and bacteriostatic nature of thyroid hormones, explain the rarity of this location. However, tuberculosis may be promoted by some factors such as advanced age, diabetes, immunosuppression (HIV) and malnutrition [5].

Clinically, very few signs can lead to the diagnosis of thyroid tuberculosis, especially as it can take the appearance of all thyroid diseases [1]. Indeed, it can be a lone nodule, a diffuse, multiheteronodular [1] or even a plunging goiter^5^. Lymphadenopathies can exist, but point rather to neoplastic lesions [3]. The presence of another tuberculous focus or even sequelae can help for diagnosis.

Sometimes, we can see an abscess organization with fistulization of the skin or a neighboring organ. We can even see compression signs such as dysphagia or laryngeal paralysis [1]. General signs can be absent. Sometimes, the only clinical sign is an unexplained mild fever [5, 6]. At the beginning of evolution, hyperthyroidism may occur subsequent to the destruction of the parenchyma and massive release of thyroid hormones. Thereafter, hypothyroidism can occur by total destruction of the gland [1]. Few cases of association of tuberculous thyroiditis and thyroid carcinoma have been reported in the literature. Hizawa and al. [7] described a case of thyroid tuberculosis associated with a pulmonary miliary tuberculosis in a patient in the postpartum period. The diagnosis was established by fine needle aspiration cytology of a palpable thyroid nodule. Despite the anti-TB treatment, the persistence of high levels of thyroglobulin and the presence of a hard thyroid nodule led to subtotal thyroidectomy with unilateral neck dissection revealing a papillary carcinoma associated with a tuberculosis of the gland. Allan and al. [8] also reported a case of tuberculosis of the thyroid lodge, occurring seven years after total thyroidectomy for medullary thyroid carcinoma.

El kohen and al. [3] reported the case of a young patient, in whom the totalization for papillary microcarcinoma found after partial thyroidectomy revealed an associated tuberculous thyroiditis. Vaishali and al. [9] described the case of a patient with nodular goiter and cervical lymphadenopathy in whom FNAC revealed a papillary carcinoma. Thyroid totalization showed the concomitant presence of thyroid tuberculosis. As in our observation, in the few cases reported in the literature of the association papillary thyroid tuberculosis and cancer, thyroid function tests were normal. The peculiar feature in our case is that the two diseases are limited to one thyroid lobe. Neither ultrasound, CT scan, nor nuclear magnetic resonance is useful to distinguish TB from other thyroid pathologies. FNAC advocated by some teams is only valuable if positive [6]. In our case, if a FNAC was performed before surgery, we could have missed the papillary carcinoma associated to the tuberculosis. Some authors suggest the possibility of diagnostic confirmation by polymerase chain reaction (PCR) after culturing glandular cell homogenate or pus issuing from a fistulous orifice [10].

The frozen section is sometimes difficult to interpret with carcinomas. In fact, only the histological study of the surgical specimen can establish a definitive diagnosis. The presence of a giant cell epithelioid granuloma with caseous necrosis is the positive diagnosis of the TT [6]. The diagnosis may also be performed by culturing the caseum and / or the surgical specimen. The other tests can be interesting to guide the diagnosis (TST, blood count and ESR) and / or find a second infectious focus (chest radiography, sputum analysis...) [1]. HIV serology may be justified depending on the context [5, 6]. The TT treatment is medical and consists of the combination of powerful anti-tuberculous drugs according to the regimen: 2RHZ/4RH. Surgical treatment helps the drainage of abscess collections, allowing diagnosis and increasing the effectiveness of medical treatment [5]. Surgery is sometimes rapidly performed in some complicated forms.

## Conclusion

Thyroid tuberculosis is a rare condition, the positive diagnosis remains histological and/or bacteriological. The association with papillary carcinoma in the same thyroid lobe is an exception. The clinical presentation of this association is variable. The treatment is medical and surgical and prognosis is usually favorable except in disseminated forms.
